# The added value of WES reanalysis in the field of genetic diagnosis: lessons learned from 200 exomes in the Lebanese population

**DOI:** 10.1186/s12920-019-0474-y

**Published:** 2019-01-21

**Authors:** Nadine Jalkh, Sandra Corbani, Zahraa Haidar, Nadine Hamdan, Elias Farah, Joelle Abou Ghoch, Rouba Ghosn, Nabiha Salem, Ali Fawaz, Claudia Djambas Khayat, Mariam Rajab, Chebl Mourani, Adib Moukarzel, Simon Rassi, Bernard Gerbaka, Hicham Mansour, Malek Baassiri, Rawane Dagher, David Breich, André Mégarbané, Jean Pierre Desvignes, Valérie Delague, Cybel Mehawej, Eliane Chouery

**Affiliations:** 1 Unité de Génétique Médicale, Faculté de Médecine, Campus De l’innovation et du sport, Université Saint-Joseph, rue de Damas, Beirut, Lebanon; 2 Service de technologie de l’information, Saint Joseph University, Beirut, Lebanon; 3 Neuropediatrics Department, Lebanese University, Beirut, Lebanon; 4 Division of Pediatrics, Hotel Dieu de France Hospital, Beirut, Lebanon; 5 Department of Pediatrics Faculty of Medicine, Saint Joseph University, Beirut, Lebanon; 6 Department of Pediatrics, Makassed General Hospital, Beirut, Lebanon; 7 Department of Otolaryngology-Head and Neck Surgery, Hotel Dieu de France Hospital, Faculty of Medicine, Saint Joseph University, Beirut, Lebanon; 8 Pediatric Neurometabolic Unit, Saint George University Medical Center, Beyrouth, Lebanon; 9 Department of Oncology, Hammoud Hospital University Medical Center, Saida, Lebanon; 10 Department of Pediatrics, Notre Dame De Secours University Hospital, Byblos, Lebanon; 11 Department of Pediatrics, Chtoura Hospital, Chtoura, Lebanon; 12 Unité de Génétique Médicale, Faculty of Medicine, Saint Joseph University, Beirut, Lebanon; 13 Institut Jérôme Lejeune, Paris, France; 14 Aix Marseille Univ, Inserm, MMG, U 1251 Marseille, France

**Keywords:** High throughput sequencing, Exome, NGS, Mutations, Genetic heterogeneity, Genetic diagnostics, Lebanon

## Abstract

**Background:**

The past few decades have witnessed a tremendous development in the field of genetics. The implementation of next generation sequencing (NGS) technologies revolutionized the field of molecular biology and made the genetic information accessible at a large scale. However, connecting a rare genetic variation to a complex phenotype remains challenging. Indeed, identifying the cause of a genetic disease requires a multidisciplinary approach, starting with the establishment of a clear phenotype with a detailed family history and ending, in some cases, with functional assays that are crucial for the validation of the pathogenicity of a mutation.

**Methods:**

Two hundred Lebanese patients, presenting a wide spectrum of genetic disorders (neurodevelopmental, neuromuscular or metabolic disorders, etc.), sporadic or inherited, dominant or recessive, were referred, over the last three and a half years, to the Medical Genetics Unit (UGM) of Saint Joseph University (USJ). In order to identify the genetic basis of these diseases, Whole Exome Sequencing (WES), followed by a targeted analysis, was performed for each case. In order to improve the genetic diagnostic yield, WES data, generated during the first 2 years of this study, were reanalyzed for all patients who were left undiagnosed at the genetic level. Reanalysis was based on updated bioinformatics tools and novel gene discoveries.

**Results:**

Our initial analysis allowed us to identify the specific genetic mutation causing the disease in 49.5% of the cases, in line with other international studies. Repeated WES analysis enabled us to increase the diagnostics yield to 56%.

**Conclusion:**

The present article reports the detailed results of both analysis and pinpoints the contribution of WES data reanalysis to an efficient genetic diagnosis. Lessons learned from WES reanalysis and interpretation are also shared.

## Background

The identification and characterization of the molecular basis of genetic disorders is crucial for the establishment of a specific diagnosis. This allows the family to benefit from an accurate genetic counseling, the patient to be aware of his disease’s prognosis and the physician to implement adequate therapeutic approaches, when possible. The last few years have seen an outstanding improvement in the field of molecular biology [1].

Indeed, the emergence of new high-throughput technologies or Next Generation Sequencing methods (NGS) such as WGS (Whole Genome Sequencing) and WES (Whole Exome Sequencing), has revolutionized the field of genetic diagnosis and made it much more efficient and affordable [2]. While WGS provides a thorough picture of the genome, WES was developed as a practical and cost effective alternative to WGS, targeting only coding regions and canonical splice sites that represent 1–1.5% of the human genome. The rationale behind this approach is that 80–85% of the mutations responsible for Mendelian diseases are located in these regions [3–5].

All these advances are driving NGS from the research field into the clinic. However, data interpretation remains the main challenge especially that thousands of variants of unknown significance are detected in each patient [6, 7].

Here we report the use of WES for genetic diagnostics purposes in 200 Lebanese patients who presented with a wide range of phenotypes, suggesting clinical and genetic heterogeneity. An initial analysis was performed followed, 2 years later, by data reanalysis in order to improve the genetic diagnostic yield. The outcome of WES in the Lebanese population is discussed and lessons learned from this study are shared.

## Methods

### Clinical samples

From January 2015 to June 2018, 200 patients with genetically heterogeneous disorders were included in our study. They were referred from different Lebanese areas, by private sector and academic physicians. All patients underwent a detailed review of their clinical history and a laboratory evaluation. The most common features of the 200 patients were global developmental delay/intellectual disability, encephalopathy, muscular weakness, failure to thrive and microcephaly.

Approval to conduct the study was obtained from the Ethics Committee of Saint Joseph University, Beirut, Lebanon. All patients, parents or legal guardians signed an informed consent for participation, sample collection and data publication. Peripheral blood was then collected from each individual enrolled in this study and DNA was extracted using the salting out method [8].

### WES analysis

Exon capture and sequencing: The exome was captured using the SureSelect Human All Exons, reagents (Agilent Inc.® Santa Clara, CA) according to the manufacturer’s standard protocol. The concentration of each library was determined using Agilent’s QPCR NGS Library Quantification Kit (G4880A). Samples were pooled prior to sequencing with a final concentration of each sample equal to 10 nM. Sequencing was performed on the Illumina HiSeq2000 platform using TruSeq v3 chemistry.

**Fig. 1:**
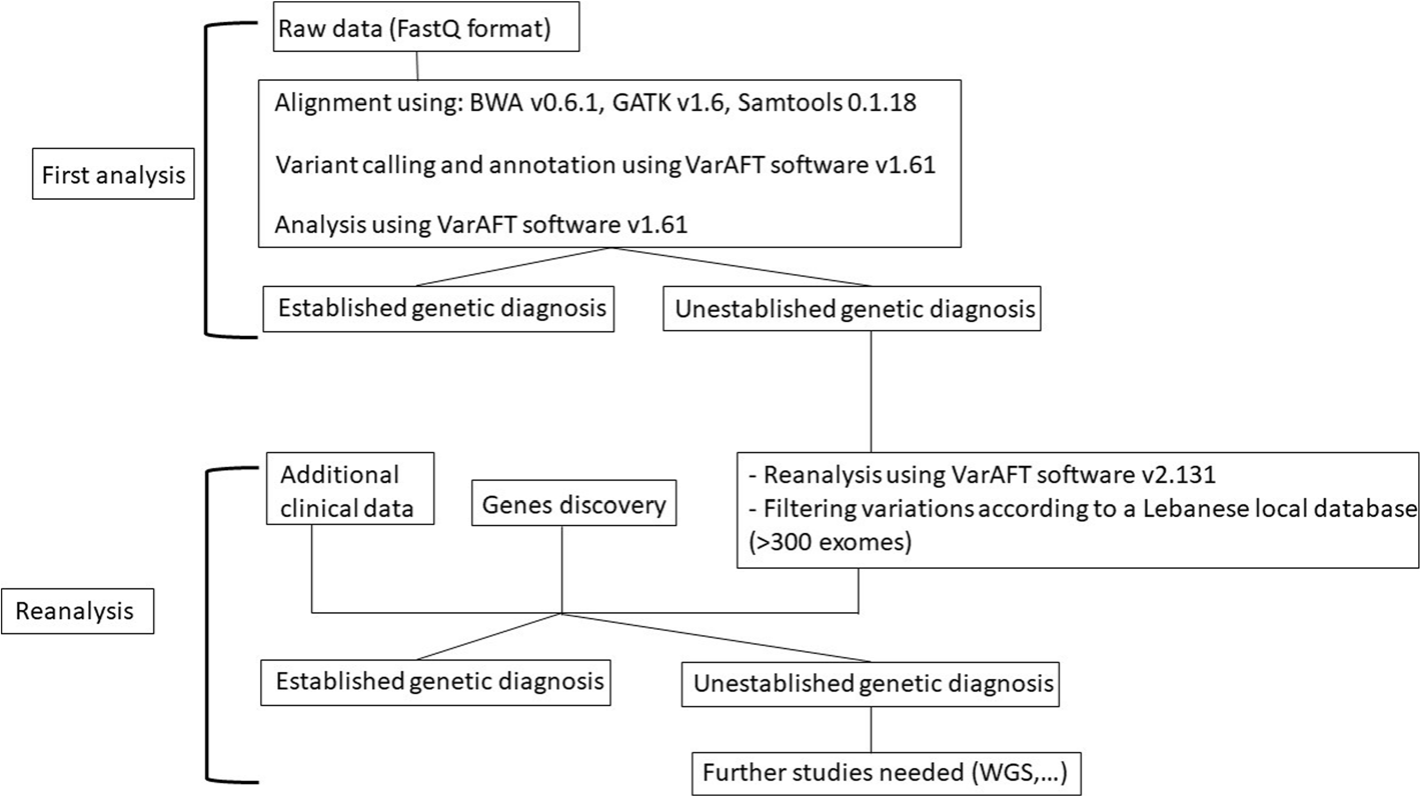
Flowchart illustrating the methodology of WES analysis carried out in this study

Mapping and alignment: Reads files (FASTQ) were generated from the sequencing platform via the manufacturer’s proprietary software. Reads were aligned to the hg19/b37 reference genome using the Burrows-Wheeler Aligner (BWA) package v0.6.1 [9]. Local realignment of the mapped reads around potential insertion/deletion (Indel) sites was carried out with the Genome Analysis Tool Kit (GATK) v1.6 [10]. Duplicate reads were marked using Picard v1.62. Additional BAM file manipulations were performed with Samtools 0.1.18 [11]. Base quality (Phred scale) scores were recalibrated using GATK’s covariance recalibration. SNP and Indel variants called using the GATK Unified Genotyper for each sample [12]. Variants were called using high stringency settings and annotated with VarAFT software 1.61 [13] containing information from dbSNP147 and ExAC (http://exac.broadinstitute.org/). For all patients who were left undiagnosed, reanalysis of vcf files was carried out using VarAFT2.131 and polymorphisms were further filtered using our in-house local database containing more than 300 exomes (Fig. 1).

In terms of functional annotation, we included only protein-altering variants, including truncating variants (stop gain/loss, start loss, or frameshift), missense variants, canonical splice-site variants, inframe indels affecting protein-coding regions, and variants within the intron–exon boundary (ten bases flanking the exonic boundaries). We focused on genotypes absent in our local control data sets. We systematically considered four different genetic models, using stratified European and African Americans in the Exon Variant Server (EVS) for minor allele frequency estimations: (a) germ-line de novo mutations, also absent in the local control populations; (b) recessive homozygous genotypes, which were heterozygous in both parents, never homozygous in controls, with a control allele frequency < 1%; (c) hemizygous X chromosome variants inherited from an unaffected heterozygous mother, with a control allele frequency < 1% and never observed in male controls or homozygous in female controls; and (d) compound heterozygous genotypes in the patient (one variant inherited from each heterozygous parent, with the two variants occurring at different genomic positions within the same gene), for which neither variant was ever homozygous in controls, and each had a control allele frequency < 1%. For the compound heterozygous genotypes, we further required study of phasing of the two variants. Genotypes meeting these criteria were referred to as “candidate genotypes,” with the genes harboring candidate genotypes referred to as “candidate genes” [14].

### Variants confirmation and segregation studies by sanger sequencing

Primers were designed using Primer 3 (http://frodo.wi.mit.edu) and OLIGOS v.9.3, and checked for specificity using BLAST (https://blast.ncbi.nlm.nih.gov/Blast.cgi). DNA sequences were obtained from UCSC and Genbank databases. Standard PCR reactions were performed using Taq DNA polymerase (Invitrogen Life Technologies, Carlsbad, CA, USA) and both strands of the resultant products were sequenced using the BigDye® Terminator v1.1 Cycle Sequencing Kit (ThermoFisher Scientific, Waltham, MA, USA) under standard conditions. The labeled products were subjected to electrophoresis on an ABI 3500 Genetic Analyzer sequencing system (ThermoFisher Scientific, Waltham, MA, USA). Electropherograms were analyzed using Sequence Analysis Software version 5.4 (ThermoFisher Scientific, Waltham, MA, USA) and compared to reference sequences using ChromasPro version 1.7.7 (Technelysium, Queensland, Australia). Nucleotide numbering reflects cDNA numbering with + 1 corresponding to the A of the ATG translation initiation codon in the reference sequences.

## Results

Over a 42-month period, 200 cases with heterogeneous genetic disorders were enrolled in this study. Among these patients, around 80% (159 samples) were younger than 18 years of age. From the latter, 150 cases were children, including the case of a fetus whose specimen was collected after a terminated pregnancy.

Patients were referred by physicians for presenting with (Table 1): neurodevelopmental disorders (in 39.5% of the cases), neuromuscular disorders (10%), metabolic and mitochondrial disorders (9.5%), renal disorders (5%), hearing disorders (3.5%), isolated epilepsy (3%), bone diseases (2%), leukodystrophy (2%), visual disorders (1%) and other rare diseases referred as “others” in all tables (24.5%). WES was performed on these patients for diagnosis purposes.

**Table 1:** Distribution of cases between disease groups with the corresponding success rate

Pathologies	Number of patients presenting the pathology	Number of patients with established molecular diagnosis	Percentage of patients with established molecular diagnosis (%)
Neurodevelopmental disorders	79	24	30.4
Neuromuscular disorders	20	17	85
Metabolic and mitochondrial disorders	19	16	84.2
Renal disorders	10	6	60
Hearing disorders	7	7	100
Epilepsy	6	4	66.7
Bone diseases	4	3	75
Leukodystrophy	4	3	75
Visual disorders	2	2	100
Others	49	30	61.2
Total	200	112	56

Our first analysis yielded a success rate of 49.5%. In other words, genetic diagnosis was established in 99 patients, after the first analysis (Table 2). In order to improve the diagnosis yield, reanalysis of WES data, generated during the first 2 years of this study, was carried out. Reanalysis was performed using new bioinformatics algorithms and was based on the newly established local database, new genes discoveries and additional clinical information for each of the undiagnosed patients. The second analysis allowed the identification of the pathogenic mutations in 13 additional cases (Table 3) corresponding to 6.5% increase in the genetic diagnosis rate, thus leading to an overall success rate of 56%. The 112 patients with established genetic diagnosis included 54 patients with autosomal dominant diseases, 49 with autosomal recessive diseases, and 9 with X-linked diseases. Different types of mutations were detected including small frameshift, nonsense, splice sites, and missense mutations.

**Table 2:**
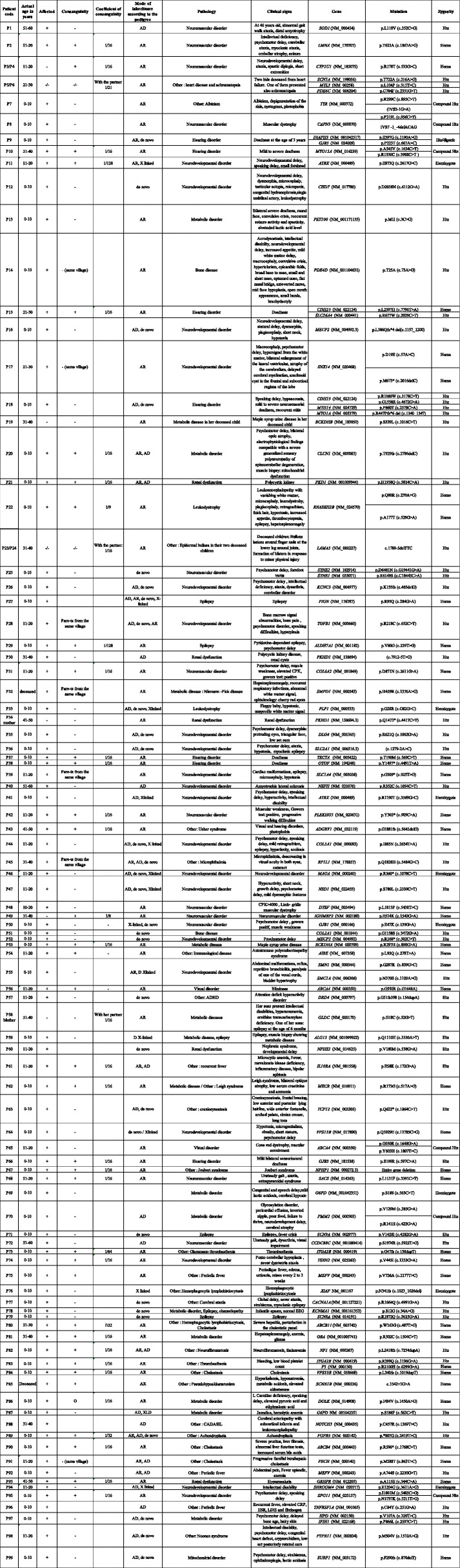
Clinical and genetic characteristics of families with established genetic diagnosis following the first analysis

*AD* Autosomal dominant, *AR* Autosomal recessive, *Htz* Heterozygous, *Homoz* Homozygous

**Table 3:**
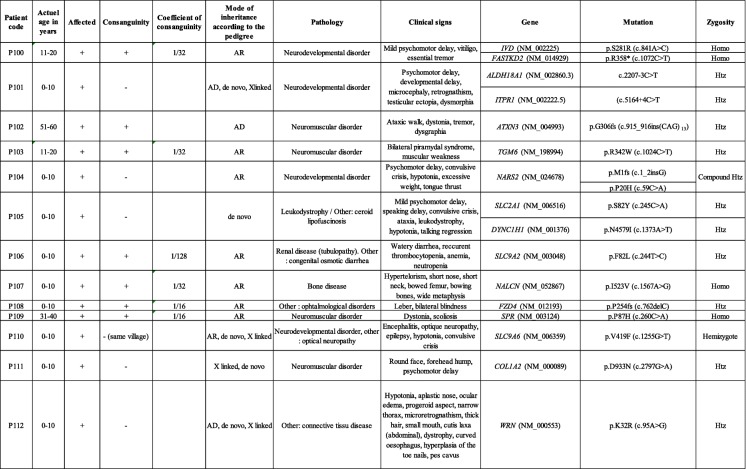
Clinical and genetic characteristics of the 13 additional cases with established genetic diagnosis after reanalysis

*AD* Autosomal dominant, *AR* Autosomal recessive, *Htz* Heterozygous, *Homoz* Homozygous.

There was a trend towards an association between the rate of positive diagnosis and the disease group. Indeed, the highest diagnosis success rate corresponds to the group of hearing and visual disorders (100%), followed by neuromuscular disorders (85%), metabolic and mitochondrial disorders (84.2%), bone diseases and leukodystrophy (75%), epilepsy (66.7%), others (61.2%), renal disorders (60%) and neurodevelopmental disorders (30.4%) (Table 1).

Of the 136 identified mutated alleles, 66.9% were novel variants at the time of diagnosis.

## Discussion

WES is a relevant, efficient and cost effective genetic diagnostic tool that allows the detection of a wide range of genetic variants including point mutations (missense and nonsense mutations), small deletions and insertions in addition to canonical splicing mutations [15]. However, linking genetic variations to diseases still represents a big challenge in many cases.

Here we report the first study on WES outcome in the Lebanese population. Exome sequencing performed in 200 cases included in our study first yielded a 49.5% overall success rate, which is concordant with other international studies [16]. This rate was variable depending on the disease group. It barely reached 30.4% in some cases such as in neurodevelopmental diseases including intellectual disabilities (ID) and autism spectrum disorders (ASD) but was extended to 100% in the case of some diseases with well-established molecular mechanisms, such as visual and hearing disorders.

In order to improve the diagnostic yield, WES raw data that was generated over the first 2 years were reanalyzed. Repeated WES analysis enabled us to establish the genetic diagnosis, for 13 additional patients who were left undiagnosed, corresponding to around 7% increase in the diagnosis rate. This positive outcome is due to many factors, among which: the development of new annotation and filtering tools (including new bioinformatics variant effect prediction logarithms), the continuous revelation of new genes in addition to the implementation at our research unit of a Lebanese WES database that allowed us to filter out all “private” Lebanese polymorphisms. Apart from these factors, the 2 years of experience in WES analysis were an asset that helped us improve our performance in data interpretation. Here we report some of the lessons learned from our study:

*Two monogenic diseases can masquerade as a single entity*: the first molecular investigation of both parents (P5 and P6) of two deceased patients presenting with heart failure and achromatopsia did not lead to any success. However, reanalysis helped to identify, in each parent, two heterozygous mutations in *MYL3* and *PDE6C*, responsible for two different conditions: heart defect (OMIM #608751) and achromatopsia (OMIM #613093), respectively. The observation, in a single patient, of multiple “hits” leading to a unique clinical presentation is nowadays becoming more and more acceptable.

*Molecular geneticists need to be aware of filtering out homozygous mutations classified, by bioinformatics logarithms, as “heterozygous uncertain” due to poor read depth*: A homozygous mutation in *PET100* in the patient P13, classified as heterozygous uncertain by our logarithm was missed in our first WES analysis. Reanalysis allowed the detection of this candidate mutation and Sanger sequencing enabled its confirmation.

*Patients from consanguineous families can still present with compound heterozygous mutations:* Patients P10 and P95, issued form consanguineous Lebanese families, were for instance shown to carry respectively two compound heterozygous mutations in *MYO15A* responsible for an autosomal recessive type of deafness (OMIM #600316) and in *SPG11* responsible for spastic paraplegia type 11 (OMIM #604360).

*De-novo mutations linked to autosomal dominant conditions can occur in patients from consanguineous families:* A de novo mutation in *GJB3* (p.S199R) involved in hearing loss was detected in patient P66 who is issued from a consanguineous Lebanese family.

*Variable Expressivity and incomplete penetrance of some diseases might mislead the choice of the filtering strategy:* Patient P94 who was expected to carry a de novo mutation, was found to have a hemizygous mutation (p.E1204G) in *SHROOM4*, inherited from his mother who shares with him milder clinical signs.

*Pathogenic mutations with a high frequency in a population exist! Filtering out common variants can be risky in the case of genetic disorders that are frequent in a population:* In patient P87 presenting with G6PD deficiency, a disease frequently seen in the Lebanese population, WES reanalysis led to the identification of a pathogenic mutation in *G6PD* (p.S188F) that is present in high frequency (greater than 1%) in several arab populations [17].

*WES has technical limitations*: Large deletions encompassing candidate genes were detected by CGH arrays in two patients (not included in the study), who were left undiagnosed by WES.

*WES coverage data might, in some cases, enable the detection of gene deletion: NPHP1* gene coverage was for instance equal to zero in patient P67 who presented with Joubert syndrome. This gene was fully covered in other patients run on the same sequencing chip. Owing to the potential involvement of the gene in the disease, CGH array was performed and a homozygous deletion encompassing the entire *NPHP1* sequence was confirmed, thus explaining the clinical presentation.

Last but not least,

*Communication between clinicians and geneticists is the ultimate key for genetic diagnostics success*: In our first WES analysis, genetic diagnostic was not established for the patient P106 who was referred to our center as presenting with Bartter syndrome. While following up on all undiagnosed cases during our WES reanalysis, a pathogenic mutation in *SLC9A2* (p.F82 L) was highlighted and communicated to the physician who re-evaluated the patient and modified the initial diagnosis to congenital chloride diarrhea, known to result from mutations in *SLC9A2* [18]*.*

On the other hand, failure of genetic diagnostics in the remaining patients can be due to several factors: i) the clinical and genetic heterogeneity of some entities: More than 1000 genes are for instance linked to ASD, thus making genetic diagnostics very challenging (https://gene.sfari.org/database/human-gene/); ii) the detection in isolated cases of private mutations whose pathogenicity is hard to be demonstrated in addition to iii) purely technical limitations associated with WES: Some bioinformatics pipelines are for example unable to detect CNVs (Copy Number Variation), rearrangements or triplet expansions. Homopolymers can for instance be miscalled, thus generating false positive variants. Furthermore, lack of coverage of coding regions (inefficient capture of regions rich in GC or bad read depth) and misalignment of reads to the reference sequence are all factors that can occur and lead to false negatives [19, 20].

## Conclusion

In conclusion, WES allowed a rapid and cost effective identification of the molecular bases of heterogeneous genetic disorders in the Lebanese population. Our study yielded an overall success rate of 56%, of which around 7% is due to data reanalysis, thus pinpointing the utility of WES reanalysis that should take into consideration the updates in bioinformatics logarithms/annotation tools, novel genetic findings in addition to the occurrence in patients of new clinical manifestations.

Undiagnosed patients need to undergo further molecular investigation. For these cases, Whole Genome Sequencing (WGS) can be performed in order to enhance coverage performance and to detect non-coding mutations (variants modifying gene expression or affecting cryptic splice sites) and structural variations including big insertions and deletions. However, this approach remains laborious and very costly.

On the other hand, current genetic diagnosis studies focuses on the protein-coding regions and ignores the vast majority of non-coding regulating elements such us non-coding RNAs. We believe that the recent advances in the fields of computational biology and experimental technology will allow a better characterization of these elements, which might enable their integration in similar studies in the future.

Altogether, the outstanding improvement in high-throughput sequencing techniques will enable the establishment of low-cost genetic diagnostics, the identification of novel genes and the elucidation of physiological mechanisms, all driving towards personalized medicine.

## Abbreviations

AD: Autosomal dominant
AR: Autosomal recessive
CGH: Comparative Genome Hybridization
CNVs: Copy number variations
NGS: Next-generation sequencing
WES: Whole-exome sequencing
WGS: Whole-genome sequencing
